# TXNRD2 (rs35934224) CT genotype and primary open-angle glaucoma: correspondence

**DOI:** 10.5935/0004-2749.20220096

**Published:** 2025-08-21

**Authors:** Pathum Sookaromdee, Viroj Wiwanitkit

**Affiliations:** 1 Private Academic Consultant, Bangkok Thailand.; 2 Honorary professor, Dr DY Patil University, Pune, India.

Dear Editor,

We would like to share ideas on “TXNRD2 (rs35934224) CT genotype as possible protective marker for primary open-angle glaucoma in a Brazilian population^(1)^.” Tenório et al. concluded that “*Our data suggest an association between TXNRD2 gene polymorphism (rs35934224) with primary open-angle glaucoma in an admixed Brazilian population*^(1)^. We agree TXNRD2 (rs35934224) CT genotype might be associated with primary open-angle glaucoma. The present report can give good data on genetic underlying background of glaucoma. In this study, Tenório et al. focused only a polymorphism.

Environmental confounding factors might be well controlled but there is a chance of effects of other genetic polymorphisms which are reported for association with primary open-angle glaucoma might still exist. Examples of those genetic polymorphisms are CDKN2B-AS1, GSTO1, GSTO2, GSTP1, GPX1OPA1, MFN1, and MFN2 polymorphisms^(2-4)^. Further studies on effects of other polymorphisms are very interesting.

## References

[1] Tenório AL, Lira RPC, Carmo RFD, Galvão Filho RP, Falcão Neto PT, Vaz RT (2021). TXNRD2 (rs35934224) CT genotype as possible protective marker for
primary open-angle glaucoma in a Brazilian population. Arq Bras Oftalmol [Internet].

[2] Sobot V, Stamenkovic M, Simic T, Jerotic D, Djokic M, Jaksic V (2021). Association of GSTO1, GSTO2, GSTP1, GPX1 and SOD2 polymorphism
with primary open angle glaucoma. Exp Eye Res..

[3] Liu S, Chen S, Niu T (2021). Genetic association between CDKN2B-AS1 polymorphisms and the
susceptibility of primary open-angle glaucoma (POAG): a meta-analysis from
21,775 subjects. Ir J Med Sci..

[4] Milanowski P, Kosior-Jarecka E, Łukasik U, Wróbel-Dudzińska D, Milanowska J, Khor CC (2021). Associations between OPA1, MFN1, and MFN2 polymorphisms and
primary open angle glaucoma in Polish participants of European
ancestry. Ophthalmic Genet.

