# Supplementary material for: TXNRD2 (rs35934224) CT genotype and primary open-angle glaucoma: correspondence
Source: Arq Bras Oftalmol. 2025 Aug 21;85(2):214–5. doi: 10.5935/0004-2749.20220096 (PMC12416629; doi:10.5935/0004-2749.20220096)
Supplement: Related texts

# Reply to “TXNRD2 (rs35934224) CT genotype and primary open-angle glaucoma: correspondence”

**Authors:** Artur Lins Tenório, Rodrigo Pessoa Cavalcanti Lira, Rodrigo Feliciano do Carmo, Roberto Pedrosa Galvão Filho, Pedro Teixeira Falcão Neto, Rinalva Tenório Vaz, Raul Emidio de Lima, André Lins Tenório, Luydson Richardson Vasconcelos

**Affiliations:** 1 Programa de Pós-graduação em Cirurgia, Universidade Federal de Pernambuco, Recife, PE, Brazil.; 2 Universidade Federal do Vale do São Francisco, Petrolina, PE, Brazil.; 3 Instituto de Olhos do Recife, Recife, PE, Brazil.; 4 Hospital de Olhos Santa Luzia, Recife, PE, Brazil.; 5 Instituto Aggeu Magalhães, Campus da Universidade Federal de Pernambuco, Recife, PE, Brazil.; 6 Faculdade de Ciências Médicas, Universidade de Pernambuco, Recife, PE, Brazil.

To the Editor:

We thank Sookaromdee and Wiwanitkit for their interest and comments on our manuscript. The authors have highlighted the importance of further studies to evaluate the effects of other polymorphisms on primary open-angle glaucoma (POAG). As published by Weinreb et al.^(1)^, glaucoma is a multifactorial disease with incompletely understood pathophysiology. Bailey et al.^(2)^ published a genome-wide association study (GWAS) describing 22 POAG-related single nucleotide polymorphisms (SNPs), of which 19 had been previously described. In addition, Bonnemaijer^(3)^ confirmed the relationship of 15 SNPs with glaucoma in the African population.

Although GWAS studies are important to identify new common polymorphisms associated with diseases, this strategy has some limitations^(4)^ that may lead to false-positive results. In addition, most studies were performed in European and North American populations; therefore, it is of fundamental importance to confirm these polymorphisms in other populations, such as the Brazilian, which has a highly admixed population. Therefore, we decided to verify the association of *TXNRD2* (rs35934224) with POAG in a Brazilian population.

As exposed in our work, we also have the conviction that further studies are needed to confirm these associations and try to better understand the disease pathophysiology to be able to propose curative therapies based on these data.

## References

[1] Weinreb RN, Leung CKS, Crowston JG, Medeiros FA, Friedman DS, Wiggs JL (2016). Primary open-angle glaucoma. Nat Rev Dis Primers..

[2] Bailey JN, Loomis SJ, Kang JH, Allingham RR, Gharahkhani P, Khor CC (2016). Genome-wide association analysis identifies TXNRD2, ATXN2 and
FOXC1 as susceptibility loci for primary open-angle
glaucoma. Nat Genet [Internet].

[3] Bonnemaijer PWM, Iglesias AI, Nadkarni GN, Sanyiwa AJ, Hassan HG, Cook C, Simcoe M, Taylor KD, Schurmann C, Belbin GM, Kenny EE, Bottinger EP, van de Laar S, Wiliams SEI, Akafo SK, Ashaye AO, Zangwill LM, Girkin CA, Ng MCY, Rotter JI, Weinreb RN, Li Z, Allingham RR, Nag A, Hysi PG, Meester-Smoor MA, Wiggs JL, Hauser MA, Hammond CJ, Lemij HG, Loos RJF, van Duijn CM, Thiadens AAHJ, Klaver CCW, GIGA StudyGroup, Eyes of Africa Genetics Consortium, NEIGHBORHOOD Consortium (2018). Genome-wide association study of primary open-angle glaucoma
in continental and admixed African populations. Hum Genet [Internet].
